# Longitudinal developmental profile of newborns and toddlers treated for spinal muscular atrophy

**DOI:** 10.1177/17562864231154335

**Published:** 2023-02-20

**Authors:** Magali Ngawa, Fabian Dal Farra, Andrei-Dan Marinescu, Laurent Servais

**Affiliations:** Neuromuscular Reference Center, Department of Paediatrics, University Hospital Liège & University of Liège, Belgium; Division of Child Neurology, Centre de Références des Maladies Neuromusculaires, Department of Pediatrics, University Hospital Liège & University of Liège, Liège, Belgium; Division of Child Neurology, Centre de Références des Maladies Neuromusculaires, Department of Pediatrics, University Hospital Liège & University of Liège, Liège, Belgium; Department of Pediatric Neurology, ‘Alexandru Obregia’ Psychiatry Hospital, Bucharest, Romania; MDUK Neuromuscular Center, Department of Paediatrics, University of Oxford, Oxford, UK; Division of Child Neurology, Centre de Références des Maladies Neuromusculaires, Department of Pediatrics, University Hospital Liège & University of Liège, 4000 Liège, Belgium

**Keywords:** Bayley, presymptomatic treatment, psychomotor development, spinal muscular atrophy

## Abstract

**Background:**

Spinal muscular atrophy (SMA) results from a loss-of-function mutation in the *SMN1* gene. SMA patients suffer progressive motor disability, although no intellectual impairments have been described. Three drugs have been recently approved by the US Food and Drug Administration (FDA) and the European Medicines Agency (EMA). These drugs result in longer life expectancy for SMA type 1 (SMA1) patients.

**Objective:**

The objective of the study was to assess longitudinally the psychomotor development of patients with SMA1 treated after the symptom onset and of patients treated presymptomatically.

**Design:**

Longitudinal, monocentric, noninterventional, prospective study.

**Methods:**

Our study included 11 SMA1 patients and seven presymptomatic SMA patients. The SMA1 patients were treated with an approved drug beginning after onset of symptoms; treatment for the presymptomatic patients was begun before symptom onset. They were longitudinally evaluated between September 2018 and January 2022 using the Bayley Scales of Infant and Toddler Development™ – Third Edition.

**Results:**

At each time point, all patients treated presymptomatically scored above those treated postsymptomatically on the motor scale. The cognitive scores of six of the seven patients treated presymptomatically were average; one patient was in the low average range. In the 11 postsymptomatically treated patients, four scored either in the low average or the abnormal range on the cognitive scale, but a positive trend was observed during the follow-up.

**Conclusion:**

A significant proportion of patients treated postsymptomatically scored below average on cognitive and communicative scales, with most significant concerns raised about the age of 1 year. Our study indicates that intellectual development should be considered as an important outcome in treated SMA1 patients. Cognitive and communicative evaluations should be performed as part of standard of care, and guidance should be provided to parents for optimal stimulation.

## Introduction

Spinal muscular atrophy (SMA) is a genetic disease with autosomal recessive inheritance. Symptoms are due to degeneration of the alpha motoneurons in the spinal cord. It was the second most common genetic cause of death in children before the introduction of new treatments.^1^ SMA is caused by a lack of SMN protein, which results from a loss-of-function mutation in the *SMN1* gene, most frequently a homozygous deletion of exon 7. Humans also have a variable number of copies of a very closely related gene, *SMN2*. The *SMN* pre-mRNA is alternatively spliced, and most mature mRNAs lack exon 7.^2^ The severity of SMA largely depends on the number of copies of *SMN2*. Patients with two copies present with the most severe and frequent form called SMA type 1 (SMA1). Symptom onset occurs before the age of 6 months, and patients are never able to sit independently. Patients with more copies of *SMN2* may develop symptoms between the age of 6 and 18 months (SMA2) or afterward (SMA3); those with SMA2 can sit autonomously but do not walk, whereas those with SMA3 are able to walk autonomously.

All forms combined, SMA affects about 1/12.000 births.^3^ This disease can cause severe disability in children and adults with significant lifelong costs.^4^ SMA patients are intellectually normal^5,6^ or even have intelligence slightly above the average, especially with respect to language.^7^ Since December 2016, three drugs have been US Food and Drug Administration (FDA) and European Medicines Agency (EMA) approved:^8^ nusinersen, an antisense oligonucleotide that modulates splicing of *SMN2* injected intrathecally 3 times per year after an initial starting dose period;^9^ onasemnogene abeparvovec, an AAV9-mediated gene therapy injected once intravenously, which provides a new copy of the gene that encodes SMN;^10^ and risdiplam, a modifier of *SMN2* splicing that is orally administered daily.^11^ These drugs have dramatically disrupted the natural course of the disease. Patients with early onset of symptoms, who without treatment seldom survive beyond the age of 2 years and therefore had never been evaluated for intellectual abnormalities, have a much longer life expectancy with treatment. This has raised the question of whether the intellectual development of patients with SMA1 is normal or not. The three drugs all have better efficacy in patients treated early, ideally before the onset of symptoms,^12^ and this has led to implementation of newborn screening for SMA in a number of countries.^13,14^

The lack of study of intellectual development in treated SMA patients with early onset of symptoms has resulted in growing use of developmental scales in the clinical trials assessing these patients over the long term (NCT 03779334). To evaluate intellectual developmental in treated patients, we longitudinally analyzed a cohort of 11 SMA1 patients and seven presymptomatic SMA patients with Bayley Scales of Infant and Toddler Development™ – Third Edition (BSID III).^15^ These patients were treated in our center over a period of 3 years. The SMA1 patients were treated with an approved drug beginning after onset of symptoms; treatment for the presymptomatic patients was begun before symptom onset.

## Methods

This was a single-site, noninterventional, prospective, observational study. Eligible for the study were all SMA1 and presymptomatic patients younger than 3 years followed in our center (*n* = 18). Patients in the study had received a molecular diagnosis of SMA. Patients with SMA2 and SMA3 were not included in this study as the number of treated patients younger than 42 months was too low and as the developmental trajectory of these patients is already well characterized. Eleven patients were treated with nusinersen, three with risdiplam, and four with onasemnogene abeparvovec. Eight patients initially treated by nusinersen were shifted to risdiplam during the study. The main criterion for treatment selection was the availability of the drug at the time the patient was diagnosed. For several patients identified during the program, nusinersen was the only option available at that time. When options were available, parents were given information about the drugs, including a written summary that can be accessed on www.beforesma.com. The decision of which drug to use was made consensually between parents and the treating physician.

Of the 18 patients included in the study, eight were identified through the Southern Belgium Newborn Screening Programme.^16^ Of these, two were clearly symptomatic at the age of diagnosis and treatment initiation, which is the case for about 40% of patients identified by NBS who have two copies of *SMN2*.^17^ Thus, we refer to these two patients as treated postsymptomatically. A patient not identified through the NBS program was screened because a sibling was affected. She is referred as presymptomatically treated, as she was actually presymptomatic at treatment initiation.

Nine patients were diagnosed because they presented with symptoms. In total, 11 patients in our cohort (nine identified per symptoms and two patients identified by NBS but symptomatic) were treated postsymptomatically.

**Figure 1.**
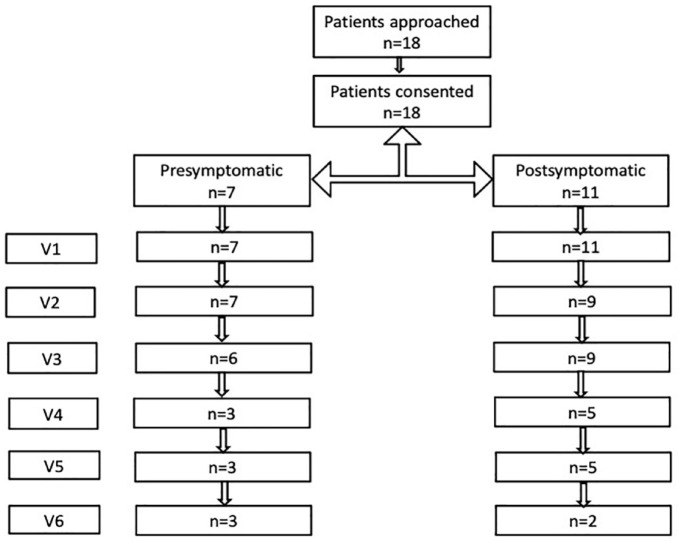
Flow chart of number of subjects enrolled and assessed over time.

We initially planned to evaluate patients every 4 months, but the COVID-19 pandemic made intervals variable at between 1 and 14 months. Patients were evaluated using the BSID III between September 2018 and 26 January 2022, which is considered as the time of last follow-up. Subjects were evaluated between 1 and 6 times (Figure 1). Testing was conducted by a neuropsychologist and a physiotherapist and had a maximum duration of 45 min. The BSID III^15^ is a developmental tool for children up to 42 months, and it consists of three scales in which patients score are compared with values observed in normally developing children: cognition (91 items), communication with two subscales: receptive (49 items) and expressive (46 items), and motor function with two subscales fine (66 items) and gross motor function (72 items).

All assessments were conducted following the BSID III manual.^15^ Patients were given a pause when deemed necessary by the investigator or the parents. The BSID III has been validated in a large control population, and normative data are available; therefore, no control population was used in this study. Data are presented as average > 85, low average 70–85, and abnormal < 70. Given the number of subjects, only nonparametric statistics were used. Final assessments for patients treated before and after the symptoms were compared using Wilcoxon–Mann–Whitney test. Correlations between continuous variables were evaluated using the Spearman correlation test. Longitudinal evolution of scores was examined using Friedman test for repeated value. All analyses were performed using SPSS v27.

## Results

In this longitudinal study, we assessed the developmental profiles of pre- and postsymptomatic patients with SMA1 using BSID-III. Patient demographics are reported in Table 1. The patients in our cohort had received a molecular diagnosis of SMA1, and those who presented with symptoms of the disease did so between 30 and 180 days old. None of the subjects in our cohort were able to sit before treatment was initiated. Throughout this article, we refer to the seven patients who began treatment before the onset of symptoms as ‘pre-symptomatic’ and to the 11 who had symptoms at the time of treatment initiation as ‘post-symptomatic’ (Figure 1). Testing was conducted by a neuropsychologist and a physiotherapist using BSID III.^15^

**Table 1.** Patient demographics.

Nr.	Sex	*SMN2* copies	SMA type	First symptoms (days)	Treatment start (days)	Treatment	Follow-up duration (in days)	Baseline CHOP-INTEND	Maximum motor function
1	M	3	PS	NA	32	N-R	1308	58	Walker
2	F	2	1	60	81	N-R	1229	23	Sitter
3	F	3	1	165	372	N-R	1219	52	Sitter
4	F	2	1	30	81	N-R	1848	22	Sitter
5	M	2	1	60	145	N-R	1726	17	Sitter
6	F	2	1	31	38	N-R	1274	42	Standing
7	M	3	1	180	488	N	870	45	Sitter
8	F	3	PS	NA	169	N	NA	60	Walker
9	M	3	PS	NA	41	OA	1066	43	Walker
10	M	2	1	60	144	OA	1019	24	Sitter
11	F	2	1	42	141	OA	1021	24	Sitter
12	F	4	PS	NA	42	R	916	44	Walker
13	F	2	1	75	118	N-R	731	22	Nonsitter
14	F	3	PS	NA	43	N	688	50	Standing
15	M	3	1	60	295	N-R	1000	5	Nonsitter
16	F	3	PS	NA	37	R	205	46	Sitter
17	M	2	1	NA	54	OA	619	/	Standing
18	F	4	PS	NA	-5	R	275	51	Sitter

N, nusinersen; NA, not applicable; N-R, nusinersen then risdiplam; OA, onasemnogene abeparvovec; PS, presymptomatic patient; SMA, spinal muscular atrophy; R, risdiplam.

Patient 18 was born at 34 weeks of gestational age and was treated at 36 weeks + 2 days of gestational age, thus day –5 in corrected age.

No presymptomatic patient was evaluated before treatment initiation. Six of the seven presymptomatic patients were first assessed between 1 week and 6 months after initiating treatment, and one was first evaluated 25 months after treatment initiation. In postsymptomatic patients, the first assessment was conducted before treatment initiation in two of 11 patients; in the other nine, the first assessment was conducted between 1 week and 22 months after the treatment initiation. Patients were followed for up to 3 years.

**Figure 2.**
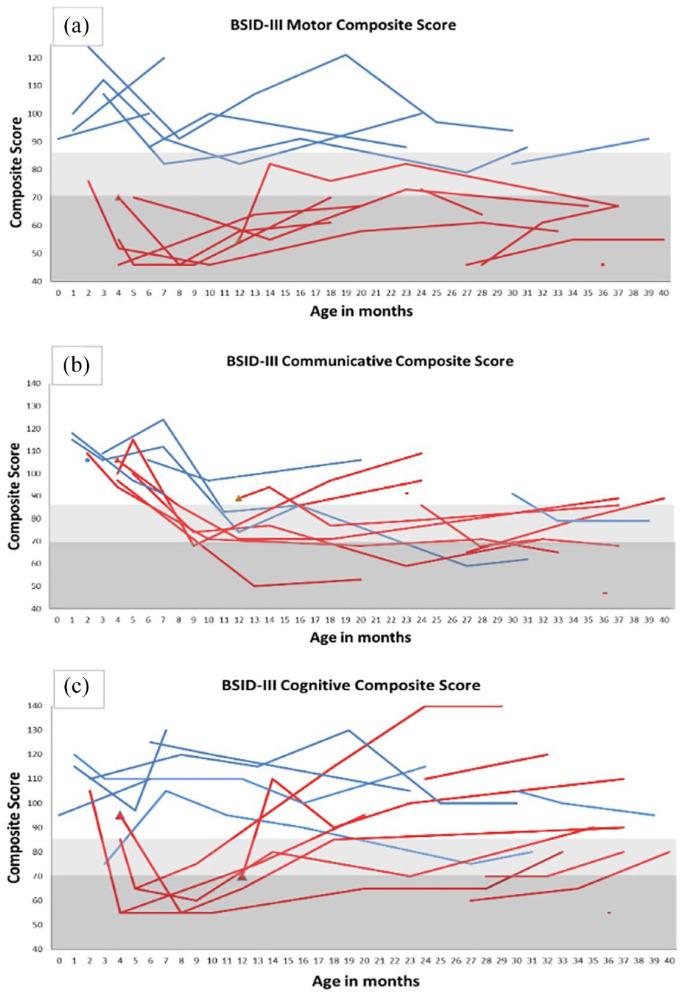
Longitudinal evolution of (a) motor, (b) communicative, and (c) cognitive BSID-III composite scores in presymptomatic (blue) and postsymptomatic (red) patients. A filled triangle indicates an evaluation performed before treatment initiation. Light gray indicates borderline score and dark gray indicates abnormal scores. One presymptomatic patient had only one assessment for communicative scale before parents refused further assessment.

At the last assessment, six out of seven presymptomatic patients scored in the average range on the motor scale, and one scored in the low average. Of 11 postsymptomatic patients, one scored in the low average, and 10 patients scored abnormal (Figure 2(a)). Four of the seven presymptomatic patients were in the average range on the communicative scale, one scored in the low average, one scored in the abnormal range, and one was not tested. Of 11 postsymptomatic patients, five scored within the average range on this scale, one scored in the low average, and five patients were in the abnormal range (Figure 2(b)).

The cognitive scores of the presymptomatic patients were in the average range for six of seven patients, and one was in the low average range. Of 11 postsymptomatic patients, seven scored in the average, three in the low average, and one was abnormal (Figure 2(c)). The patient in the abnormal range, patient 15, presented with focal seizures at the age of 2 years and severe cognitive impairment with poor contact; brain magnetic resonance imaging (MRI) of this patient was normal. Visual inspection of the patient trajectories suggests that the cognitive score passes through a minimum from 6 to 12 months and then increases.

In an unplanned analysis, we compared the cognitive score at the last assessment with the value at the assessment around 9 months for patients with available data (*n* = 6); this confirmed that there was an improvement in the cognitive score at the end of the study (*p* = 0.01). We found significant correlations at the final assessment between motor and cognition (*r* = 0.528; *p* = 0.043) and between cognition and communication (*r* = 0.728; *p* = 0.002) but not between motor and communication (*r* = 0.433; *p* = 0.107) subscores.

In another unplanned exploratory analysis, we compared maximal values of subscores achieved during the study with the subscores at the last assessment for patients with two copies of *SMN2* to all other patients. Those treated both pre- and postsymptomatically were included. Only for the gross motor score was the maximal (*p* = 0.037) or the last (*p* = 0.030) value achieved during the study significantly lower in patients with two copies. No difference between patients with 2 *SMN2* copies and other patients was observed for cognitive or communicative subscore.

## Interpretation

In this longitudinal study, we assessed the developmental profiles of SMA1 patients treated pre- and postsymptomatically using BSID-III. Presymptomatic patients had better motor outcomes than postsymptomatic patients. All patients treated before symptom onset scored above all postsymptomatic patients in the motor subscore. This is in line with the data from clinical trials^18–20^ and all data accumulated from real-world experience: Patients with SMA1 have the potential to improve on a motor perspective even if treated late,^21^ but shorter disease duration before treatment initiation is associated with better motor outcomes^12^ and lower medical and social costs.^22^

Visual inspection of patient trajectories for communicative subscore suggests that this aspect of cognition should be carefully followed up in all patients. A recent study reported speech difficulties in postsymptomatic patients, mostly due to articulation.^23^ Nevertheless, the trajectories of patients reported here should be interpreted with caution given the variabilities in subscores within subjects. We found a strong correlation between communication and cognitive composite scores in our patients, and a weaker correlation between motor and cognitive scores. This is consistent with previous findings in several conditions, including cerebral palsy.^24^ Phones, tablets, computers, and TVs are used in SMA1 patients to minimize the impact of invasive care and to distract subjects with lower mobility. The number of hours spent by some of these patients watching phones or tablets has not yet been formally reported but is a concern shared by several treating physicians. The overuse of screens in younger infants has been associated with slower language development.^25^

In the 11 postsymptomatically treated patients, six scored either in the low average or the abnormal range in the cognitive subscore, and eight were below average in the communicative composite subscore. This result is not in line with previous reports of neuropsychological profiles of untreated patients with SMA, who have been described as within the norm or even slightly above.^5–7^ Most research so far has been conducted in SMA2/3 patients, however. Separation anxiety has been reported as a potential neuropsychological trait in older SMA2/3 patients.^26^ A recent report suggests that SMA3 patients are below average in visuospatial abilities, executive functions, and language as compared with healthy controls.^27^

The reports of neuropsychological profiles of subjects with SMA1 are scarce (for a recent review, see Masson *et al.*^28^) and only concern untreated patients.^29^ Some patients with very severe and early presentation reportedly have cerebral malformations.^30^ The absence of cognitive follow-up data in SMA1 subjects is mainly due to the fact that before the first disease-modifying treatments, the survival of SMA1 patients was usually limited to 2 years,^29^ and it was not a priority to assess cognition. The increased survival rate in patients treated with nusinersen,^9^ onasemnogene abeparvovec,^10^ or risdiplam^11^ has led to an increased number of patients surviving with SMA1.^30^ Our data suggest that cognitive development should be evaluated further in treated patients. The situation in SMA may be similar to that in Pompe disease. Adult- and juvenile-onset Pompe patients have no cognitive abnormalities, but in the congenital form that led to death in 100% cases before the age of 1 year, treated patients present with a mild cognitive dysfunction.^31,32^ Cognitive impairment in treated SMA1 patients may be the result of brain hypoperfusion^33^ or low expression of SMN protein in cortical neurons,^29,34^ which is not entirely corrected by disease-modifying treatments, especially in very severe cases or patients with low copy numbers of *SMN2*. In mouse models of SMA, brain abnormalities have been observed.^35^

Our study suffers from several limitations, the first being the small number of patients enrolled. Our population of presymptomatic patients is too small to allow conclusions regarding cognitive development. Nevertheless, we observed that all but one patient had normal cognitive profiles. Further observation and the long-term follow-up of the patient in the borderline zone will be needed to strengthen this initial reassuring observation. A comparison of patients treated pre- and postsymptomatically was not possible due to the small number of subjects. More data are thus needed, not only from clinical trials, but also from the real-world experience, including patients treated later than in clinical trials in which the inclusion age is generally before the age of 6 or 7 months. This exploratory study was not powered to demonstrate a difference between drugs and no formal comparison was conducted. The second limitation was the limited follow-up due to the COVID pandemic. Another limitation, which is an issue with all studies of young subjects, is the difficulty ensuring the cooperation of young children in neuropsychological assessments. Our study also suggests that cognitive assessment scores differ significantly with time, even in a monocentric study during which assessments were conducted by a single rater.

Despite these limitations, our study indicates that intellectual development should be considered as an important outcome in treated SMA1 patients. The effects on intellectual development could also constitute an important factor in treatment choice when enough data are available on outcomes after treatment with approved medications. Intellectual development follow-up should be included in standard of care, and guidance should be provided to parents for optimal intellectual stimulation of very weak infants.

## Conclusions

Our study provides the first prospective longitudinal data of intellectual development in presymptomatically and postsymptomatically treated SMA1 patients. Our data raise concerns regarding the non-motor development especially in this latter group. Further research on a broader sample and longer follow-up are needed to confirm these data and measure the long-term impact of this possible cognitive impairment.

## Supplemental Material

sj-doc-1-tan-10.1177_17562864231154335 – Supplemental material for Longitudinal developmental profile of newborns and toddlers treated for spinal muscular atrophy Click here for additional data file. Supplemental material, sj-doc-1-tan-10.1177_17562864231154335 for Longitudinal developmental profile of newborns and toddlers treated for spinal muscular atrophy by Magali Ngawa, Fabian Dal Farra, Andrei-Dan Marinescu and Laurent Servais in Therapeutic Advances in Neurological Disorders

## Declarations

**Ethics approval and consent to participate:** The research was approved by the Ethical Comittee of CHR Citadelle (NUB B412201838505, N/Ref: 1779) and all parents gave written informed consent for participation.

**Consent for publication:** Not applicable.

**Availability of data and materials:** Access to data can be request by any qualified investigator.
